# Comparative Analysis of Complete Chloroplast Genomes of *Rubus* in China: Hypervariable Regions and Phylogenetic Relationships

**DOI:** 10.3390/genes15060716

**Published:** 2024-05-31

**Authors:** Yufen Xu, Yongquan Li, Yanzhao Chen, Longyuan Wang, Bine Xue, Xianzhi Zhang, Wenpei Song, Wei Guo, Wei Wu

**Affiliations:** 1 Department of Horticulture and Landscape Architecture, Zhongkai University of Agriculture and Engineering, Guangzhou 510225, China; yufen_xu@163.com (Y.X.); yongquanli@zhku.edu.cn (Y.L.); yenzhaochan@163.com (Y.C.); wanglongyuan@zhku.edu.cn (L.W.); xuebine@zhku.edu.cn (B.X.); zhangxianzhi@zhku.edu.cn (X.Z.); songwenpei@zhku.edu.cn (W.S.); wuei06@gmail.com (W.W.); 2 Coconut Research Institute, Chinese Academy of Tropical Agricultural Sciences, Wenchang 571339, China

**Keywords:** *Rubus*, chloroplast genome, phylogeny, mutation hotspot, infrageneric relationship

## Abstract

With more than 200 species of native *Rubus*, China is considered a center of diversity for this genus. Due to a paucity of molecular markers, the phylogenetic relationships for this genus are poorly understood. In this study, we sequenced and assembled the plastomes of 22 out of 204 Chinese *Rubus* species (including varieties) from three of the eight sections reported in China, i.e., the sections *Chamaebatus*, *Idaeobatus*, and *Malachobatus*. Plastomes were annotated and comparatively analyzed with the inclusion of two published plastomes. The plastomes of all 24 *Rubus* species were composed of a large single-copy region (LSC), a small single-copy region (SSC), and a pair of inverted repeat regions (IRs), and ranged in length from 155,464 to 156,506 bp. We identified 112 unique genes, including 79 protein-coding genes, 29 transfer RNAs, and four ribosomal RNAs. With highly consistent gene order, these *Rubus* plastomes showed strong collinearity, and no significant changes in IR boundaries were noted. Nine divergent hotspots were identified based on nucleotide polymorphism analysis: *trnH-psbA*, *trnK-rps16*, *rps16-trnQ-psbK*, *petN-psbM*, *trnT-trnL*, *petA-psbJ*, *rpl16* intron, *ndhF-trnL*, and *ycf1*. Based on whole plastome sequences, we obtained a clearer phylogenetic understanding of these *Rubus* species. All sampled *Rubus* species formed a monophyletic group; however, sections *Idaeobatus* and *Malachobatus* were polyphyletic. These data and analyses demonstrate the phylogenetic utility of plastomes for systematic research within *Rubus*.

## 1. Introduction

*Rubus* L. is a large and diverse genus in the family Rosaceae. Besides being among the most important genetic resources of berry fruits [1], *Rubus* species also have great potential as medicines [2,3,4] and in ecological restoration [5,6]. There are at least 750 to 1000 species of *Rubus* worldwide, mainly distributed in northern temperate areas [7]. In China, there are ca. 204 species, accounting for 97% of Asian species [8,9]; thus, China is considered a key center of *Rubus* diversity, with 138 endemic taxa, mainly distributed in Southwest China [10]. Due to frequent hybridization, polyploidization, and apomixis [11,12,13], the classification of *Rubus* is challenging [13]. Among different classification systems, Focke’s system with 12 subgenera and 22 sections was the first to be widely accepted [14,15,16]. More recent systems, such as the Flora of China, recognizing eight sections and 24 subsections, were applied to Chinese *Rubus* species [7,17], with sections *Idaeobatus* (83 species, 11 subgroups) and *Malachobatus* (86 species, 13 subgroups) being the most diverse [17]. Extensive studies on morphology [18,19], palynology [20,21], and cytology [22,23,24] notwithstanding, prevailing classifications were still controversial. Notably, the monophyly of some recognized sections has not been supported [25,26,27,28], and the placement of certain species remains uncertain [11]. Phylogenetic analyses with few genetic makers were insufficient to obtain highly confident phylogenetic trees [11], and resultant phylogenetic trees were poorly resolved. Therefore, a broader array of suitable genetic markers is urgently needed to clarify phylogenetic relationships, essential for the efficient development and utilization of the *Rubus*.

With a typical quarter-ring structure, plant plastomes are usually composed of two inverted repeat regions (IRs) separated by a large single-copy region (LSC) and a small single-copy region (SSC). Plastomes sizes of most seed plants range between 120 and 160 kb [29], with occasional significant enlargement or reduction, such as in *Pelargonium transvaalense* (242 kb, KM527900), *Actinidia cylindrica* (224 kb) [30], *Carnegiea gigantea* (113 kb) [31], and *Hordeum spontaneum* (114 kb, KC912688). In most cases, the contraction, expansion, or loss of IRs leads to changes in plastome size [32]. The gene content and order of angiosperm plastomes are usually very conservative, but rearrangements can be found in some families, including Leguminosae [33], Campanulaceae [34], Geraniaceae [35], and Oleaceae [36]. These rearrangements can be associated with the loss of genes or introns [37], IR expansion, inversion [38], and repetitive sequence expansion [39] or transposable elements (TEs) [40,41]. With the merits of moderate substitution rates, high homology, and often maternal inheritance nature, plastomes are now extensively used for phylogenetic analyses across diverse plant lineages [42,43,44]. For example, Zhang et al. [45] reconstructed the phylogeny of Rosaceae based on the plastome data, with most nodes being well resolved and well supported. Li et al. [46] recovered high phylogenetic resolution along the backbone of the tribe Potentilleae within the Rosaceae family based on plastid phylogenomics.

In this study, we used short reads from Illumina sequencing to assemble and annotate 22 plastomes of Chinese *Rubus* species for genome characterization and phylogenetic analysis. Our objectives were to (1) characterize the composition, structure, and sequence variation of *Rubus* plastome; (2) identify variation hotspots in these plastomes; and (3) explore the potential utility of plastomes for the phylogenetic reconstruction of *Rubus*.

## 2. Materials and Methods

### 2.1. Plant Material and DNA Sequencing

Twenty-two *Rubus* species representing 20 subsections of three sections (*Idaeobatus*, *Malachobatus*, and *Chamaebatus*) were collected from Yunnan, Sichuan, Chongqing, Guangxi, Guangdong, and Hainan provinces in China (Table S1). The identification of each species was verified twice by Bine Xue and Longyuan Wang, and corresponding voucher specimens were deposited in the herbarium of Sun Yat-sen University (SYS) (Table S1). Young, healthy leaves of each collection were dried with silica gel, and their DNA was extracted using a modified CTAB method. Prior to sequencing, the integrity of the DNA samples was checked by gel electrophoresis and the purity was evaluated with the NanoDrop 1000 spectrophotometer (Thermo Scientific, Wilmington, DE, USA) with a ratio of absorbance at 260 nm/280 nm ca. 1.8 and finally measured (>20 ng·μL^−1^) with a Qubit fluorometer (Invitrogen, Carlsbad, CA, USA). A NEBNex^®^t Ultra^TM^ II DNA Library Prep Kit (New England Biolabs) was used to construct a library with an insert size of 350 bp, which was sequenced with at least 20-fold coverage on the Illumina HiSeq X™ Ten Sequencing System (San Diego, CA, USA), with 150 bp paired-end reads.

### 2.2. Assembly, Annotation, and Visualization of Chloroplast Genome

By using the de novo assembler NOVOPlasty 3.6 [47] with default parameters, the 22 *Rubus* plastomes were assembled with 5 million raw reads after the removal of adapters and further annotated with *Rubus leucanthus* (MK105853) as a reference with the package Plann [48] with default parameters. Further rRNA annotations were implemented in GeSeq [49] (https://chlorobox.mpimp-golm.mpg.de/geseq.html (accessed on 20 December 2019)) with default parameters, and all annotation information was checked by Sequin v.9.50. After manual correction, plastome circle maps were obtained from the online web program OGDRAW (https://chlorobox.mpimp-golm.mpg.de/OGDraw.html (accessed on 31 May 2020)).

### 2.3. Genome Composition, Structural, and Sequence Variation Analysis

Two *Rubus* plastomes (*R. cochinchinensis* (MN913339) and *R. leucanthus* (MK105853)) and five plastomes from other genera of Rosaceae (*Fragaria vesca* (JF345175), *Fragaria pentaphylla* (NC034347), *Rosa multiflora* (NC039989), *Pyrus pyrifolia* (NC015996), and *Malus hupehensis* (NC040170)) were downloaded and comparatively analyzed with the 22 newly sequenced platsomes. Genome sizes, structural boundaries, gene compositions, and GC contents were counted in MEGA X [50] using default parameters for the 24 plastomes. Plastome collinearity analyses were performed and visualized with the Mauve tool [51] packaged in Geneious v.10.0.9, and the expansion or contraction of the IR boundary was visualized by the online software IRscope [52] (https://irscope.shinyapps.io/irapp/ (accessed on 12 May 2020)) using default parameters.

The plastomes of 24 *Rubus* species were compared and analyzed with *R. leucanthus* (MK105853) as a reference with Shuffle-LAGAN packaged in mVISTA [53]. The program MAFFT 7 [54] with default parameters was used to align the complete plastomes, LSC, SSC, and IRs of the24 *Rubus* species, and the corresponding numbers of mutation sites and information sites were estimated with MEGA X [50] using default parameters.

Plastome nucleotide polymorphisms (*Pi*) among the 24 plastomes were estimated by DnaSP v.6 [55] with a window length of 600 bp and a step size of 200 bp. The nine fragments with the highest *Pi* values were selected as variation hotspots among the 24 *Rubus* species, and the locations of these fragments were determined based on the annotation information.

### 2.4. Phylogenetic Analysis

The complete plastomes of 22 newly sequenced *Rubus* species and 5 other *Rubus* species from GenBank (*R. cochinchinensis* (MN913339), *R. crataegifolius* (MG189543), *R. takesimensis* (MG972806), *R. leucanthus* (MK105853), and *R. coreanus* (MH992398)) were sampled for phylogenetic analyses. Five representative species (*Fragaria vesca* (JF345175), *F. pentaphylla* (NC034347), *Rosa multiflora* (NC039989), *Pyrus pyrifolia* (NC015996), and *Malus hupehensis* (NC040170)) from four other Roseaceae genera were treated as outgroups. In addition, alignments of the nine selected fragments harboring mutation hotspots for the 27 *Rubus* species and one outgroup species (*F. vesca*) were constructed.

Prior to phylogenetic reconstruction, alignment partition schemes and substitution model determinations were implemented by using the program PartitionFinder v 2.1.1 [56] with default parameters. Following Ma’s partition scheme strategies for plastomes [57], we divided the plastome as follows: a. partition0, unpartitioned; b. partition2, coding and non-coding regions; c. partition3, LSC, IRs, SSC; partition6, rRNA, tRNA, non-coding regions, codon 1,2,3 of coding regions. The best partition schemes and corresponding substitution models were determined among the four partition schemes by maximum likelihood estimation or Akaike information criterion (AIC). Based on these partitions and corresponding substitution modes, maximum likelihood (ML) phylogenetic tree estimation was implemented in RAxML 8.2.12 [58] with 1000 bootstrap analyses using default parameters. Bayesian analysis (BI) was performed by using MrBayes 3.2.7 [59] with 1,000,000 generations and a sample frequency of 1000 generations until the average standard deviation of split frequency values was <0.01. The first 25% of samples were discarded as “burn-in”, and the remaining samples were summarized to construct a 50% majority-rule consensus tree. The convergence of MCMC for MrBayes analysis was diagnosed using Tracer 1.7.1 [60] with an effective sample size (ESS) of over 200. All phylogenetic trees were visualized using the program in FigTree v1.4.4.

## 3. Results

### 3.1. Characteristics of Chloroplast Genomes

**Figure 1:**
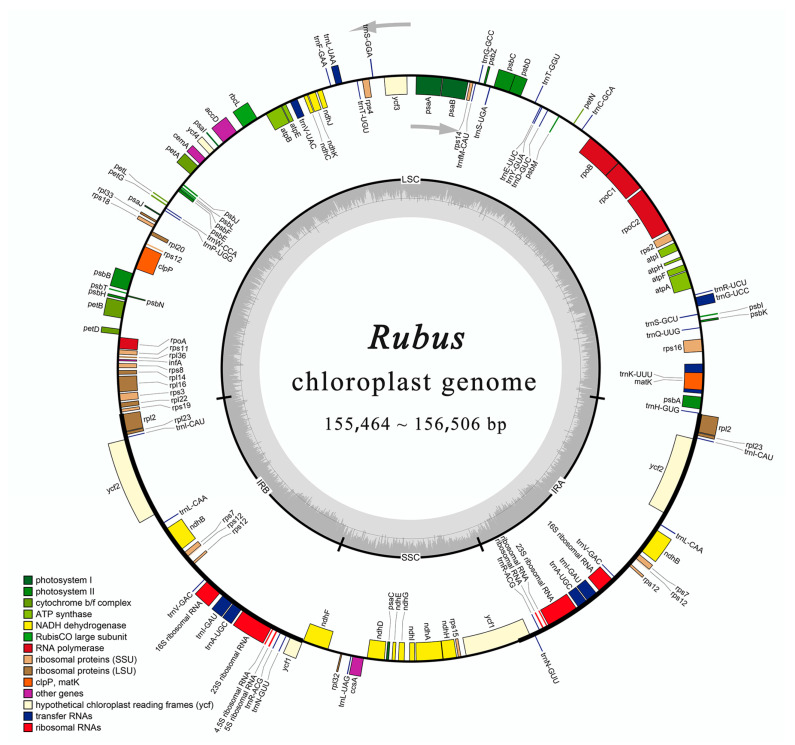
Overview of circos map for the *Rubus* chloroplast genomes. Genes in the large circle are transcribed clockwise, genes outside the large circle are transcribed counterclockwise, and dark gray in the small circle corresponds to GC content. LSC, large single-copy region; SSC, small single-copy region; IRA, IRB, inverted repeat region. Different functional genes are distinguished by color.

We sequenced and annotated the complete chloroplast genomes of 22 *Rubus* species (Table 1). All plastomes of these species contain the four typical structural regions including a large single-copy region, two inverted repeat regions, and a small single-copy region (Figure 1). Their genome size ranged between 155,464 bp (*R. pileatus*) and 156,506 bp (*R. pectinaris*) (Table 1). The LSC length varied from 84,847 bp (*R. pileatus*) to 86,214 bp *(R. pectinaris*), accounting for 54.58–55.09% of the plastome length. The SSC length ranged between 18,481 bp (*R. innominatus*) and 18,875 bp (*R. xanthocarpus*), accounting for 11.89–12.10% of the plastome length. The length of the IR (single) ranged from 25,737 (*R. peltatus*) to 25,997 (*R. pileatus*), accounting for 16.45–16.72% of the plastome length. The length ratio of each structural region to the total length was relatively stable in 24 *Rubus* species, and plastome size variation was relatively small. With an average IR region length of 18,778 bp and an average SSC region length of 25,801 bp, the variation among these species was less than 400 bp for both regions. The total chloroplast genome length, averaging 156,000 bp, showed significant variation across the 24 *Rubus* species, ranging from 6 to 1042 bp. In addition, the total length and the length of the three structural regions of the *Rubus* plastomes were similar to those species in the same subfamily (Rosoideae) (*Fragaria vesca*, *F. pentaphylla*, and *Rosa multiflora*), but quite different from members of subfamily Amygdaloideae (*Pyrus pyrifolia* and *Malus hupehensis*).

We identified 112 unique genes, including 79 protein-coding genes, 29 tRNAs, and four rRNAs in 24 *Rubus* plastid genomes, with the total number of genes ranging between 129 and 131 (Table 1). Numbers of both tRNAs and rRNAs were conserved across the Rosaceae, but those of protein-encoding genes exhibited significant variation ranging between 83 (*Pyrus pyrifolia*) and 90 (*Rosa multiflora*), while our *Rubus* species consistently had 85 protein-encoding genes. Protein-encoding genes were classified into four functional categories: self-replication (58), photosynthesis (45), other functions (5), and unknown functions (4) (Table S2). In the IR region, the composition of four rRNAs, seven tRNAs (*trnA-UGC*, *trnI-CAU*, and *trnI-GAU*, etc.), and six protein-coding genes (*rps7*, *rpl2*, *ndhB*, etc.) were common, and, sometimes, one end of trans-spliced gene *rps12 or ycf1* was observed among the 24 *Rubus* species. For protein-encoding genes with introns, two introns were observed in *clpP* and *ycf3* and 14 genes had only one intron.

GC contents among the 24 *Rubus* species were analyzed, and the average GC content of the plastomes ranged between 36.91% (*R. peltatus*) and 37.30% (*R. innominatus*), and no significant bias was observed. However, obvious differences existed in different regions, i.e., IR (42.79%), LSC (35.05%), and SSC (31.15%). Furthermore, this same pattern was observed in the other five Rosaceae species.

### 3.2. Comparative Analysis of Chloroplast Genomes and Identification of Divergence Hotspots

**Figure 2:**
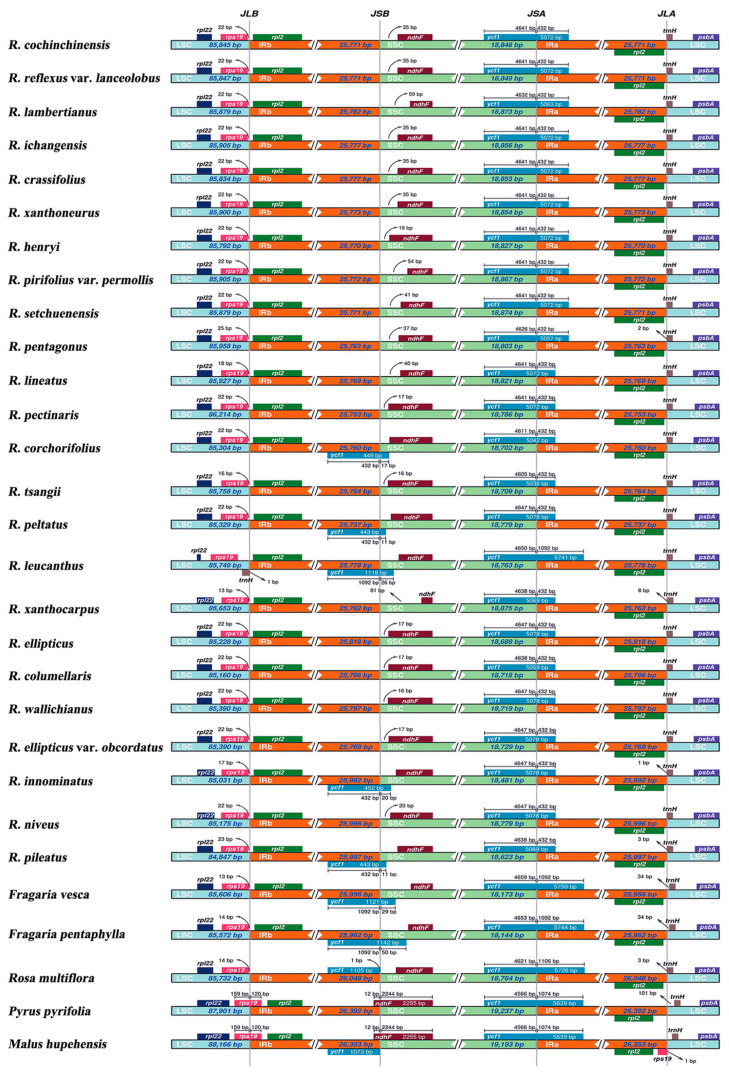
Comparison of the boundaries of LSC, IR, and SSC among chloroplast genomes of 24 *Rubus* species and 5 other Rosaceae species. JLB, junction between LSC and IRB; JSB, junction between SSC and IRB; JSA, junction between SSC and IRA; JLA, junction between LSC and IRA.

Collinearity analysis demonstrated no genome rearrangements among 24 *Rubus* species (Figure S1). In addition, the boundaries of IRA and IRB regions were relatively stable among these species (Figure 2). For instance, the LSC-IRB boundary was mainly located between *rps19* and *rpl2* (except for in *R. leucanthus*), with a distance of 13–25 bp between *rps19* and the adjacent boundary. The boundary between IRB and SSC of most *Rubus* species was close to the gene *ndhF* of the SSC region, except for five species (*R. corchorifolius*, *R. peltatus*, *R. leucanthus*, *R. innominatus*, and *R. pileatus*), where it was located in *ycf1* with a length of 11 bp to 26 bp in the SSC region. The SSC–IRA boundaries were all inside *ycf1*, with lengths varying from 4605 bp (*R. tsangii*) to 4650 bp (*R. leucanthus*) in the SSC region and with lengths from 432 bp (excluding 1092 bp of *R. leucanthus*) in the IRA region. Except for *R. pentagonus*, *R. xanthocarpus*, *R. innominatus*, and *R. pileatus*, the IRA–LSC boundary of other *Rubus* species was adjacent to *trnH* in the LSC region. Overall, the structural borders of Rosoideae species (*F. vesca*, *F. pentaphylla*, and *Rosa multiflora*) more closely resembled those of the *Rubus* species than those of two Amygdaloideae species (*P. pyrifolia* and *M. hupehensis*).

**Figure 3:**
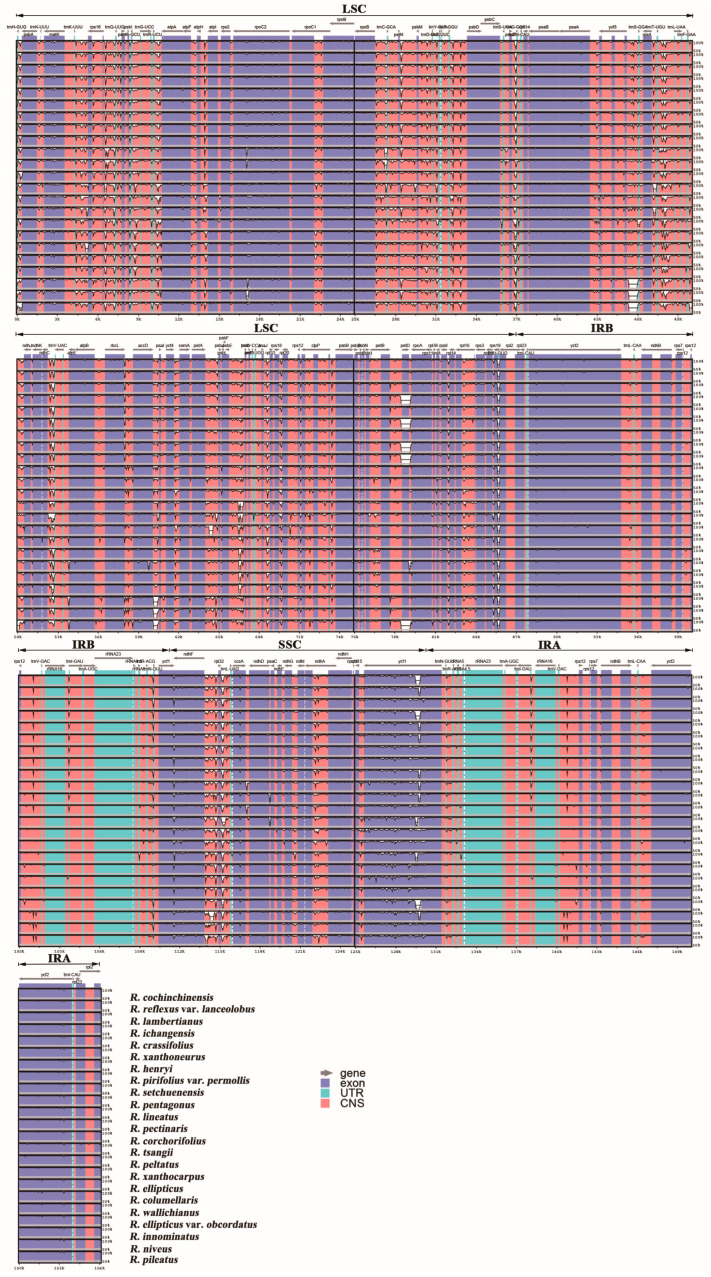
Comparative analysis of chloroplast genome sequences in 24 *Rubus* species. R. leucanthus was chosen as the reference genome; the gray arrow indicates the direction of the gene. UTR, untranslated region; CNS, non-coding sequence. The y-axis represents from 50% to 100% consistency.

**Figure 4:**
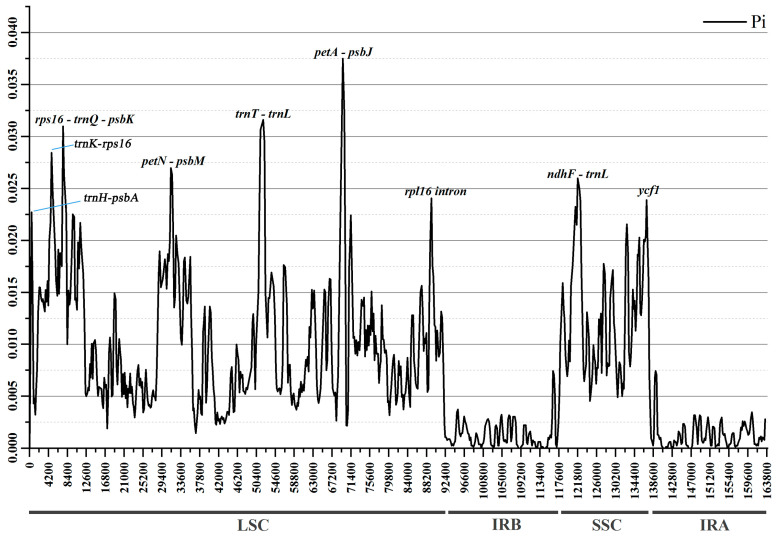
Nucleotide polymorphism analysis of chloroplast genomes in 24 *Rubus* species. The *x*-axis denotes the coordinates of the chloroplast genome and the *y*-axis represents the polymorphisms measured with *Pi*.

By using mVISTA, chloroplast genome sequences of 23 *Rubus* species were compared with reference to *R. leucanthus* annotations (Figure 3). Overall, the IR region exhibited the least variation (0.91%), much less than that of LSC (6.88%) or SSC (8.53%) (Table 2). As expected, the variable proportions of coding genes were significantly less than those of non-coding genes (introns and intergenic spacers) (Table 2). The nucleotide polymorphism (*Pi*) of plastomes among 24 *Rubus* species varied from 0 to 0.0375 (Figure 4), and the IR regions, with a mean *Pi* value < 0.005, showed the least differentiation. Based on *Pi* values, nine highly variable regions were selected (*Pi* value > 0.0225), including seven gene spacers (*trnH-psbA*, *trnK-rps16*, *rps16-trnQ-psbK*, *petN-psbM*, *trnT-trnL*, *petA-psbJ*, *ndhF-trnL*), one gene intron (*rpl16* intron), and one protein-coding gene (*ycf1*). Among them, *trnH-psbA* exhibited the highest level of polymorphism (*Pi* = 0.0565) and a parsimony information site proportion of 8.63%; *rps16-trnQ-psbK* displayed the highest variation in site proportion (17.62%).

### 3.3. Phylogenetic Analysis

**Figure 5:**
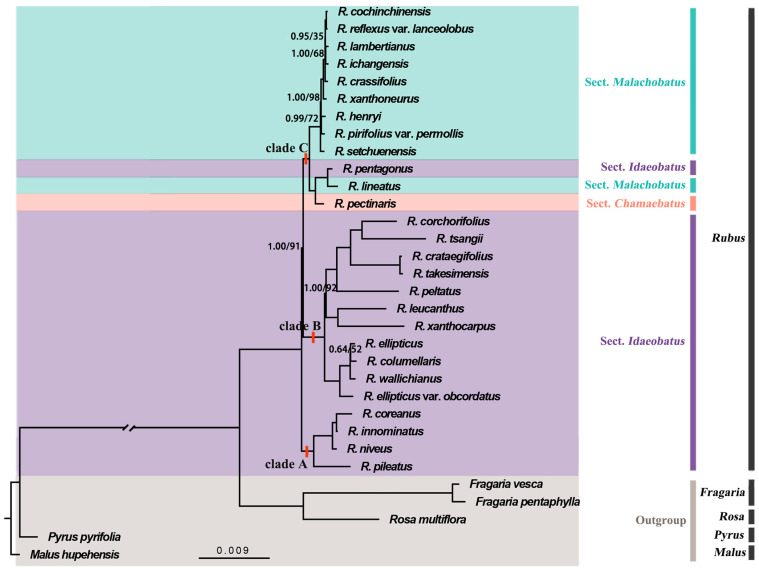
The Bayesian inference (BI) and maximum likelihood (ML) tree for *Rubus* based on the complete plastome. The number on the branch is BI posterior probability (PP)/ML bootstrap (BS); branches without numbers indicate nodes with 1.00/100 support values.

With an increased number of partitions, decreased AIC or increased maximum likelihood supported the scheme partition 6 (Table S3); thus, we divided the plastome alignment into rRNA, tRNA, non-coding regions, and codons 1, 2, and 3 of the coding regions, respectively. For each partition, GTR + I + R was selected as the best-fit substitution model. From these datasets, the tree topologies estimated by the Bayesian inference (BI) method and maximum likelihood (ML) method were consistent (Figure 5). All *Rubus* species form a monophyletic clade (PP = 1.00, ML BS = 100%) as a sister to the *Fragaria* and *Rosa* species (PP = 1.00, ML BS = 100%). The *Rubus* species split into three clades: clade A consisted of only four *Rubus* species, sister to the remaining *Rubus* species; the remaining species diverged into two distinctive sister clades (clade B and clade C). Species in clades A and B all belonged to the sect. *Idaeobatus*. The species in clade C were further divided into two subclades, one assigned to sect. *Malachobatus* and the other with three species belonging to sections *Malachobatus*, *Idaeobatus*, and *Chamaebatus*, respectively (Figure 5). The monophyly of sects. *Idaeobatus* and *Malachobatus* were therefore not supported, unless *R. pentagonus* was misassigned at the sectional level.

**Figure 6:**
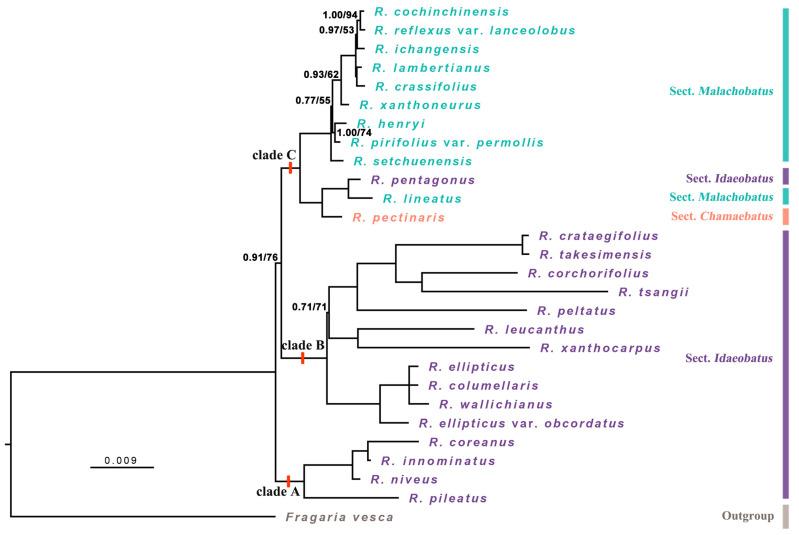
The Bayesian inference (BI) and maximum likelihood (ML) tree for *Rubus* based on nine highly variable cpDNA makers. The number on the branch is BI posterior probability (PP)/ML bootstrap (BS); branches without numbers indicate nodes with 1.00/100 support values.

ML and BI analyses were also conducted based on the dataset of the nine mutation hotspots in *Rubus* species. The phylogenetic trees based on this dataset were generally consistent with those obtained from whole plastomes, with somewhat decreased support for some nodes or position shifts for some species (Figure 6).

## 4. Discussion

### 4.1. Variation in Plastome Size and Gene Composition

The gene content of plastomes is conservative, typically involving 100 to 120 unique genes [61]. In these *Rubus* species, we identified 112 unique genes and 129 to 131 total genes, with the differences attributed to the copy numbers of *ycf1* and *trnH*-GUG. Among these, two full copies of the *ycf1* gene were only observed in five species (*R. pileatus*, *R. innominatus*, *R. leucanthus*, *R. peltatus*, and *R. corchorifolius*), with only one copy in the others. Two copies of *ycf1*, one complete and one pseudogene truncated by the IRB-SSC boundary [62], have been reported in Nelumbonaceae [63], Salicaceae [64], and Brassicaceae [65], and some have even been lost completely in some plants [66]. In 19 of the 24 *Rubus* plastomes, one copy of *ycf1* in the IRB region has been lost, but the random distribution of these species across the phylogenetic tree suggests that these events give no clear phylogenetic signal.

Of 29 Rosaceae species, two copies of *trnH-GUG* located in the LSC region were only observed in *R. leucanthus.* This duplication is common in monocotyledons (e.g., Orchidaceae, Poaceae) and some basic angiosperms (Magnoliales and Chloranthales) [67]. Mardanov et al. [68] considered that the expansion from the IR region to the LSC region might be attributed to the additional copy of *trnH-GUG*, representing an ancestral pattern in basal angiosperms. Wang et al. [69] found that the karyotypes of Chinese *Rubus* resembled those of the basic angiosperms. Hence, *Rubus* may be relatively primitive within the Rosaceae.

### 4.2. Variation and Divergence of Rubus Plastomes

So far, the expansion or contraction of IR boundaries and gene loss have been attributed to plastome size variations [70,71], and, in some taxa, these changes were phylogenetically informative [67]. Beyond the *Rubus* species, the IR boundaries expanded at *rps19* and *ndhF* genes in two other species in the subfamily Amygdaloideae, and the same expansion of IR boundaries also occurred in five species of *Prunus* in the same subfamily [72]. Therefore, we speculate that the IR boundary changes were responsible for the plastome size difference between *Rubus* and the subfamily Amygdaloideae. The plastid genome of most plants has a non-coding sequence ranging from 0 to 30 bp between the IRA–LSC boundary and the 3′ end of *trnH-GUG* [73]. However, such non-coding sequences expanded significantly and showed high similarity to mitochondrial gene sequences in some taxa, for instance, Apiales species (>200 bp) including *Petroselinum* (345 bp) [73]. We also observed a non-coding sequence ranging between one and eight bp at this position in *R. pentagonus*, *R. xanthocarpus*, *R. innominatus*, and *R. pileatus*, but it showed no phylogenetic signal.

The GC content was associated with DNA stability; the higher the GC content, the lower the mutation rates [74]. The GC content of IR, LSC, and SSC among *Rubus* species decreased in turn, which was well in accordance with the variation levels among the three regions for the *Rubus* species. In our study, variation indices, including the mutation site proportion, parsimony information site proportion, nucleotide polymorphisms, and differentiation levels, all showed the same trend among the three regions [75].

### 4.3. Development of Chloroplast DNA Markers

Chloroplast DNA markers have been widely used in *Rubus* phylogenetic analyses, e.g., protein-coding genes, such as *ndhF*, *rbcL*, and *rpl16*, and intergenic regions *trnL-trnF* and *trnS-trnG* [27]. However, most of these markers have shown limitations for phylogenetic reconstruction in *Rubus* and other Rosaceae [76,77]. In contrast, by using nine screened hyper-mutation fragments, we obtained a well-resolved phylogenetic tree comparable to one based on whole plastome sequences, and these nine markers might be useful for phylogenetic analyses in other groups of Rosaceae. Some of our recommended fragments, i.e., *trnH-psbA* [78], *trnT-trnL* [79], *petA-psbJ* [80], and *rpl16* intron [81] have been widely applied in other plants, and *ycf1a* or *ycf1b* have even been recommended as candidates for core DNA barcodes [82].

### 4.4. Phylogenetic Analysis

Due to frequent hybridization, apomixis, and polyploidy, the classification and phylogeny of *Rubus* have long been controversial [11,12,13,28]. By using whole chloroplast genomes, the resolution of the maternal phylogenetic tree can be improved significantly over those based on only a few fragments. Our phylogenetic analyses showed that *Rubus* is monophyletic and more closely related to *Fragaria* and *Rosa* than to *Pyrus* and *Malus*, consistent with another recent plastid phylogenomic study of Rosaceae [45]. Our phylogenetic analyses showed that sections *Idaeobatus* and *Malachobatus* as currently recognized were polyphyletic, consistent with previous studies of sect. *Idaeobatus* [83,84]. However, more taxon sampling will be required before proposing any new infra-generic classification.

Species in sect. *Idaeobatus* mainly have compound leaves and those in sect. *Malachobatus* mainly bear simple leaves, implying that compound leaves might be ancestral in *Rubus*. In previous studies, the relationships among three taxa, *R. ellipticus*, *R. ellipticus* var. *obcordatus*, and *R. wallichianus*, were controversial [24,85,86]. Our phylogenetic analysis revealed that *R. ellipticus* has a more distant relationship with *R. wallichianus* and *R. ellipticus* var. *obcordatus*, lending support for the recognition of *R. ellipticus* var. *obcordatus* at the species level, which will require a broader investigation.

In our study, we examined six species from section *Idaeobatus* and three from section *Malachobatus*, which overlapped with those studied by Carter et al. (2019) [28]. Our findings show a divergence in the relationship of *R*. *ichangensis* and *R*. *lambertianus* within section *Malachobatus*, unlike Carter et al. (2019), who identified them as sister species based on nuclear loci. Additionally, we observed inconsistencies in the phylogenetic relationship for *R*. *lineatus* within *Malachobatus* and for *R*. *calophyllus* and *R*. *pentagonus* within *Idaeobatus* when comparing chloroplast genomes with nuclear loci. The remaining six species examined in both studies showed that relationships among *R. niveus*, *R. coreanus*, and *R. innominatus* in our clade A are congruent with the chloroplast phylogeny reported in the earlier study [28], yet conflict with their nuclear phylogeny. Conversely, the alignment of *R. ellipticus* and *R. crataegifolius* in our clade B matches with both their chloroplast and nuclear loci findings [28]. Due to extensive natural hybridization within *Rubus*, the matrilineal chloroplast genome is difficult to explain via reticular evolution. In subsequent studies, the nuclear genome data with biparental inheritance and detailed genetic information can be applied to explore the complex evolutionary history of *Rubus* including reticular evolution, hybrid infiltration, and polyploidization.

## 5. Conclusions

In this study, we assembled and annotated the complete chloroplast genomes of 22 Chinese *Rubus* species. Comparative plastid genome analysis revealed that gene order and content were conserved among the *Rubus* species. Differences in genome size among them were small and typically related to the length variations of the LSC region. There were slight differences in the total number of genes between a few *Rubus* species. Plastid genome structures were stable, and there was no obvious expansion or contraction observed at the boundaries of the two IR regions. The composition and order of the chloroplast genes were also conservative, with high collinearity. Phylogenetic trees were constructed based on the whole chloroplast genome dataset, and the combined nine hyper-variable regions dataset strongly supported that *Rubus* species in this study formed a monophyletic group with three main clades, but both sections *Idaeobatus* and *Malachobatus* are polyphyletic. This study reaffirms the great potential of plastid genomes to resolve biosystematic relationships among *Rubus* species.

## Figures and Tables

**Table 1:** Characteristics of chloroplast genomes of 24 *Rubus* species and 5 other species in Rosaceae.

Species	Accession Number	Length (bp)				Number of Genes (Unique)				GC Content (%)			
		Total	LSC	SSC	IR	Total	PCGs	tRNA	rRNA	Total	LSC	SSC	IR
*R. cochinchinensis* *	MN913339	156,235	85,845	25,771	18,848	129 (112)	85 (79)	36 (29)	8 (4)	37.18	35.12	31.23	42.78
*R. reflexus* var. *lanceolobus*	MT576937	156,238	85,847	25,771	18,849	129 (112)	85 (79)	36 (29)	8 (4)	37.18	35.12	31.24	42.78
*R. lambertianus*	MT576938	156,316	85,879	25,782	18,873	129 (112)	85 (79)	36 (29)	8 (4)	37.16	35.11	31.18	42.78
*R. ichangensis*	MT576943	156,315	85,905	25,777	18,856	129 (112)	85 (79)	36 (29)	8 (4)	37.16	35.09	31.20	42.78
*R. crassifolius*	MT576949	156,241	85,834	25,777	18,853	129 (112)	85 (79)	36 (29)	8 (4)	37.17	35.12	31.18	42.78
*R. xanthoneurus*	MT576942	156,300	85,900	25,773	18,854	129 (112)	85 (79)	36 (29)	8 (4)	37.17	35.12	31.20	42.78
*R. henryi*	MT576954	156,159	85,792	25,770	18,827	129 (112)	85 (79)	36 (29)	8 (4)	37.18	35.11	31.23	42.80
*R. pirifolius* var. *permollis*	MT576950	156,316	85,905	25,772	18,867	129 (112)	85 (79)	36 (29)	8 (4)	37.15	35.09	31.15	42.79
*R. setchuenensis*	MT576955	156,295	85,879	25,771	18,874	129 (112)	85 (79)	36 (29)	8 (4)	37.17	35.11	31.22	42.80
*R. pentagonus*	MT576944	156,287	85,958	25,763	18,803	129 (112)	85 (79)	36 (29)	8 (4)	37.16	35.09	31.20	42.77
*R. lineatus*	MT576952	156,286	85,927	25,769	18,821	129 (112)	85 (79)	36 (29)	8 (4)	37.14	35.08	31.19	42.75
*R. pectinaris*	MT576953	156,506	86,214	25,753	18,786	129 (112)	85 (79)	36 (29)	8 (4)	37.12	35.01	31.25	42.77
*R. corchorifolius*	MT576951	155,526	85,304	25,760	18,702	130 (112)	86 (79)	36 (29)	8 (4)	37.05	34.91	30.90	42.83
*R. tsangii*	MT576948	155,995	85,758	25,764	18,709	129 (112)	85 (79)	36 (29)	8 (4)	37.01	34.80	31.04	42.86
*R. peltatus*	MT576945	155,582	85,329	25,737	18,779	130 (112)	86 (79)	36 (29)	8 (4)	36.91	34.69	30.80	42.81
*R. leucanthus* *	MK105853.1	156,068	85,749	25,778	18,763	131(112)	86 (79)	37 (29)	8 (4)	37.03	34.91	30.98	42.77
*R. xanthocarpus*	MT576946	156,052	85,653	25,762	18,875	129 (112)	85 (79)	36 (29)	8 (4)	37.06	34.94	30.98	42.83
*R. ellipticus*	MT576935	155,553	85,228	25,818	18,689	129 (112)	85 (79)	36 (29)	8 (4)	37.13	35.04	31.13	42.76
*R. columellaris*	MT576939	155,470	85,160	25,796	18,718	129 (112)	85 (79)	36 (29)	8 (4)	37.14	35.05	31.06	42.79
*R. wallichianus*	MT576941	155,703	85,390	25,797	18,719	129 (112)	85 (79)	36 (29)	8 (4)	37.12	35.01	31.05	42.80
*R. ellipticus* var. *obcordatus*	MT576940	155,657	85,390	25,769	18,729	129 (112)	85 (79)	36 (29)	8 (4)	37.15	35.04	31.11	42.83
*R. innominatus*	MT576947	155,496	85,031	25,992	18,481	130 (112)	86 (79)	36 (29)	8 (4)	37.30	35.24	31.37	42.79
*R. niveus*	MT576936	155,946	85,175	25,996	18,779	129 (112)	85 (79)	36 (29)	8 (4)	37.26	35.20	31.30	42.81
*R. pileatus*	MT576956	155,464	84,847	25,997	18,623	130 (112)	86 (79)	36 (29)	8 (4)	37.27	35.18	31.41	42.79
*Fragaria vesca* *	JF345175.1	155,691	85,606	25,956	18,173	130 (112)	85 (78)	37 (30)	8 (4)	37.21	35.12	31.11	42.81
*Fragaria pentaphylla* *	NC_034347.1	155,640	85,572	25,962	18,144	129 (111)	85 (78)	36 (29)	8 (4)	37.25	35.17	31.13	42.82
*Rosa multiflora* *	NC_039989.1	156,592	85,732	26,048	18,764	135 (117)	90 (83)	37 (30)	8 (4)	37.23	35.18	31.31	42.74
*Pyrus pyrifolia* *	NC_015996.1	159,922	87,901	26,392	19,237	126 (110)	83 (76)	35 (30)	8 (4)	36.58	34.29	30.40	42.64
*Malus hupehensis* *	NC_040170.1	160,065	88,166	26,353	19,193	129 (112)	84 (78)	37 (30)	8 (4)	36.55	34.22	30.38	42.70

LSC, large single-copy region; SSC, small single-copy region; IR, inverted repeat region; PCGs, protein-coding genes; tRNA, transfer RNA; rRNA, ribosome RNA; *, downloaded from GenBank.

**Table 2:** Summaries of variable information for 24 *Rubus* chloroplast genomes.

Region	Aligned Length (bp)	No. of VCs	VC Proportion (%)	No. of PICs	PICs (%)	*Pi*
cp genome	163,713	8392	5.13	3052	1.86	0.0074
LSC	91,588	6305	6.88	2295	2.51	0.0102
SSC	19,581	1670	8.53	591	3.02	0.0127
IR	52,250	475	0.91	152	0.29	0.0014
Coding region	92,068	2696	2.93	919	1.00	0.0046
Non-coding region	77,398	5777	7.46	2100	2.71	0.0121
*trnH-psbA*	386	61	15.80	34	8.81	0.0515
*trnK-rps16*	1399	195	13.94	83	5.93	0.0296
*rps16-trnQ-psbK*	1632	282	17.28	122	7.48	0.0248
*petN-psbM*	1427	195	13.67	71	4.98	0.0250
*trnT-trnL*	1577	274	17.37	99	6.28	0.0266
*petA-psbJ*	1185	135	11.39	77	6.50	0.0373
*rpl16 intron*	1040	116	11.15	52	5.00	0.0194
*ndhF-trnL*	2335	347	14.86	127	5.44	0.0260
*ycf1*	5790	482	8.32	165	2.85	0.0144

VCs, variable characters; PICs, parsimony informative characters; *Pi*, nucleotide polymorphism.

## Data Availability

We deposited the raw reads and chloroplast genome assemblies and annotations in NCBI’s SRA and GenBank database (https://www.ncbi.nlm.nih.gov/ (accessed on 11 January 2021)) under accession numbers SAMN15763296~SAMN15763317 and MT576935~MT576956, respectively.

## Supplementary Materials

The following supporting information can be downloaded at https://www.mdpi.com/article/10.3390/genes15060716/s1. Figure S1: Collinearity analysis of complete chloroplast genomes among 24 *Rubus* species. A long bar structure with a consistent color indicates local collinear block (LCBs), the short bar structure below represents the annotation information of the fragment, and the short red bar structure represents rRNA. Table S1: The classification and collection information of the studied *Rubus* species. List of the studied *Rubus* taxa, their taxonomic classification, herbarium voucher information, leaf type, locality, and GenBank accession numbers. Table S2: The genes in the *Rubus* chloroplast genome. Table S3: Comparison of partitioning strategies used for sequence alignments of *Rubus* and other Rosaceae.

## References

[1] Duan J., Tang H.R., Wang X.R., Li L. (2006). Application of genetic markers on identification of *Rubus* resources. Chin. Agric. Sci. Bull..

[2] Li W.L., He S.A., Gu Y. (2000). An outline on the utilization value of Chinese bramble (*Rubus* L.). J. Wuhan Bot. Res..

[3] Xuan J.H., Zhang C.Y., Meng X.J., Liu C.J. (2006). Research progress on the development and utilization of *Rubus* germplasm resources. North. Hortic..

[4] Han J., Liu J.W. (2009). Advance in studies on biological activities for *Rubus*. Chin. Wild Plant Resour..

[5] Marques A.P.G.C., Moreira H., Rangel A.O.S.S., Castro P.M.L. (2009). Arsenic, lead and nickel accumulation in *Rubus* ulmifolius growing in contaminated soil in Portugal. J. Hazard. Mater..

[6] Yang T.T. (2013). Researches progress and exploitation of *Rubus corchorifolius*. J. Sichuan For. Sci. Technol..

[7] Lu L.D. (1983). A study on the genus *Rubus* of China. Acta Phytotaxon. Sin..

[8] Li W.L., Zhao W.J. (1993). Studies on *Rubus* resources in Qinling-Bashan Mountain area. J. Plant Resour. Environ..

[9] Lu L.T., Boufford D.E., Wu Z.Y., Raven P.H., Hong D.Y. (2003). Rubus L.. Flora of China.

[10] Gu Y. (1992). *Rubus* L. resources and its utilization. J. Plant Resour. Environ..

[11] Alice L.A., Campbell C.S. (1999). Phylogeny of *Rubus* (Rosaceae) based on nuclear ribosomal DNA internal transcribed spacer region sequences. Am. J. Bot..

[12] Alice L.A. (2002). Evolutionary relationships in *Rubus* (Rosaceae) based on molecular data. Acta Hortic..

[13] Sochor M., Vašut R.J., Sharbel T.F., Trávníček B. (2015). How just a few makes a lot: Speciation via reticulation and apomixis on example of European brambles (*Rubus* subgen. Rubus, Rosaceae). Mol. Phylogenetics Evol..

[14] Focke W.O. (1910). Monographiae Generis Rubi Prodromus Pars I. Species Ruborum.

[15] Focke W.O. (1911). Monographiae Generis Rubi Prodromus Pars II. Species Ruborum.

[16] Focke W.O. (1914). Monographiae Generis Rubi Prodromus Pars III. Species Ruborum.

[17] Yu T.T., Lu L.T., Yu T.T., Lu L.T., Ku T.C., Kuan K.C., Li C.L. (1985). Rubus L. Rosaceae. Flora Reipublicae Popularis Sinicae.

[18] Abbate G., Bonacquisti S., Scassellati E. (2002). Morphological study of three taxa of the genus *Rubus* L. sect. Rubus (Rosaceae) in western central Italy. Plant Biosyst..

[19] Tomlik-Wyremblewska A., Zieliński J., Guzicka M. (2010). Morphology and anatomy of blackberry pyrenes (*Rubus* L., Rosaceae) Elementary studies of the European representatives of the genus *Rubus* L. Flora.

[20] Kasalkheh R., Jorjani E., Sabouri H., Habibi M., Sattarian A. (2017). Pollen morphology of the genus *Rubus* L. subgenus *Rubus* (Rosaceae) in Iran. Nova Biol. Reper..

[21] Xiong X., Zhou X., Li M., Xu B., Deng H., Yu Q., Gao X.F. (2019). Pollen morphology in *Rubus* (Rosaceae) and its taxonomic implications. Plant Syst. Evol..

[22] Thompson M.M. (1997). Survey of chromosome numbers in *Rubus* (Rosaceae: Rosoideae). Ann. Mo. Bot. Gard..

[23] Amsellem L., Chevallier M.H., Hossaert-McKey M. (2001). Ploidy level of the invasive weed *Rubus alceifolius* (Rosaceae) in its native range and in areas of introduction. Plant Syst. Evol..

[24] Wang Y., Wang X., Chen Q., Zhang L., Tang H., Luo Y., Liu Z. (2015). Phylogenetic insight into subgenera *Idaeobatus* and *Malachobatus* (*Rubus*, Rosaceae) inferring from ISH analysis. Mol. Cytogenet..

[25] Yang J.Y., Pak J. (2006). Phylogeny of Korean *Rubus* (Rosaceae) based on ITS (nrDNA) and *trnL/F* intergenic region (cpDNA). J. Plant Biol..

[26] Yang J., Yoon H., Pak J. (2012). Phylogeny of Korean *Rubus* (Rosaceae) based on the second intron of the LEAFY gene. Can. J. Plant Sci..

[27] Zhang L., Wang X.R., Wang Y., Chen Q., He W. (2014). Research progress of molecular phylogenetic analyses based on DNA sequence data in *Rubus* L. (Rosaceae). Acta Bot. Boreali-Occident. Sin..

[28] Carter K.A., Liston A., Bassil N.V., Alice L.A., Bushakra J.M., Sutherland B.L., Mockler T.C., Bryant D.W., Hummer K.E. (2019). Target Capture Sequencing Unravels Rubus Evolution. Front. Plant. Sci..

[29] Palmer J.D., Bogorad L., Vasil I.K. (1991). Plastid chromosomes: Structure and evolution. The Molecular Biology of Plastids.

[30] Ma X., Liu H. (2019). The complete chloroplast genome sequence of *Actinidia cylindrica* C. F. Liang. Mitochondrial DNA Part B.

[31] Sanderson M.J., Copetti D., Burquez A., Bustamante E., Charboneau J.L.M., Eguiarte L.E., Kumar S., Lee H.O., Lee J., McMahon M. (2015). Exceptional reduction of the plastid genome of saguaro cactus (*Carnegiea gigantea)*: Loss of the ndh gene suite and inverted repeat. Am. J. Bot..

[32] Cheng H., Ge C.F., Zhang H., Qiao Y.S. (2018). Advances on chloroplast genome sequencing and phylogenetic analysis in fruit trees. J. Nucl. Agric. Sci..

[33] Cai Z., Guisinger M., Kim H., Ruck E., Blazier J.C., McMurtry V., Kuehl J.V., Boore J., Jansen R.K. (2008). Extensive reorganization of the plastid genome of *Trifolium subterraneum* (Fabaceae) is associated with numerous repeated sequences and novel DNA insertions. J. Mol. Evol..

[34] Cosner M.E., Raubeson L.A., Jansen R.K. (2004). Chloroplast DNA rearrangements in Campanulaceae: Phylogenetic utility of highly rearranged genomes. BMC Evol. Biol..

[35] Chumley T.W., Palmer J.D., Mower J.P., Fourcade H.M., Calie P.J., Boore J.L., Jansen R.K. (2006). The complete chloroplast genome sequence of *Pelargonium × hortorum*: Organization and evolution of the largest and most highly rearranged chloroplast genome of land plants. Mol. Biol. Evol..

[36] Lee H.L., Jansen R.K., Chumley T.W., Kim K.J. (2007). Gene relocations within chloroplast genomes of *Jasminum* and *Menodora* (Oleaceae) are due to multiple, overlapping inversions. Mol. Biol. Evol..

[37] Jansen R.K., Cai Z.Q., Raubeson L.A., Daniell H., DePamphilis C.W., Leebens-Mack J., Müller F.K., Guisinger-Bellian M., Haberle R.C., Hansen A.K. (2007). Analysis of 81 genes from 64 plastid genomes resolves relationships in angiosperms and identifies genome-scale evolutionary patterns. Proc. Natl. Acad. Sci. USA.

[38] Zhao Y.M., Yang Z.Y., Zhao Y.P., Li X.L., Zhao Z.X., Zhao G.F. (2019). Chloroplast genome structural characteristics and phylogenetic relationships of Oleaceae. Chin. Bull. Bot..

[39] Haberle R.C., Fourcade H.M., Boore J.L., Jansen R.K. (2008). Extensive rearrangements in the chloroplast genome of *Trachelium caeruleum* are associated with repeats and tRNA genes. J. Mol. Evol..

[40] Cosner M.E., Jansen R.K., Palmer J.D., Downie S.R. (1997). The highly rearranged chloroplast genome of *Trachelium caeruleum* (Campanulaceae): Multiple inversions, inverted repeat expansion and contraction, transposition, insertions/deletions, and several repeat families. Curr. Genet..

[41] Iorizzo M., Senalik D., Szklarczyk M., Grzebelus D., Spooner D., Simon P. (2012). De novo assembly of the carrot mitochondrial genome using next generation sequencing of whole genomic DNA provides first evidence of DNA transfer into an angiosperm plastid genome. BMC Plant Biol..

[42] Maliga P. (2004). Plastid transformation in higher plants. Annu. Rev. Plant Biol..

[43] Wang X.Q., Song W.W., Xioa J.J., Li C.Q., Liu Z.H. (2019). Phylogeny of Myrtales and related groups based on chloroplast genome. Guihaia.

[44] Gitzendanner M.A., Soltis P.S., Yi T.S., Li D.Z., Soltis D.E. (2018). Chapter Ten—Plastome phylogenetics: 30 years of inferences into plant evolution. Adv. Bot. Res..

[45] Zhang S.D., Jin J.J., Chen S.Y., Chase M.W., Soltis D.E., Li H.T., Yang J.B., Li D.Z., Yi T.S. (2017). Diversification of Rosaceae since the Late Cretaceous based on plastid phylogenomics. New Phytol..

[46] Li Q.Q., Zhang Z.P., Wen J., Yu Y. (2024). Plastid phylogenomics of the tribe Potentilleae (Rosaceae). Mol. Phylogenetics Evol..

[47] Dierckxsens N., Mardulyn P., Smits G. (2017). NOVOPlasty: De novo assembly of organelle genomes from whole genome data. Nucleic Acids Res..

[48] Huang D.I., Cronk Q.C. (2015). Plann: A command-line application for annotating plastome sequences. Appl. Plant Sci..

[49] Tillich M., Lehwark P., Pellizzer T., Ulbricht-Jones E.S., Fischer A., Bock R., Greiner S. (2017). GeSeq—versatile and accurate annotation of organelle genomes. Nucleic Acids Res..

[50] Kumar S., Stecher G., Li M., Knyaz C., Tamura K. (2018). MEGA X: Molecular evolutionary genetics analysis across computing platforms. Mol. Biol. Evol..

[51] Darling A.C., Mau B., Blattner F.R., Perna N.T. (2004). Mauve: Multiple alignment of conserved genomic sequence with rearrangements. Genome Res..

[52] Amiryousefi A., Hyvönen J., Poczai P. (2018). IRscope: An online program to visualize the junction sites of chloroplast genomes. Bioinformatics.

[53] Frazer K.A., Pachter L., Poliakov A., Rubin E.M., Dubchak I. (2004). VISTA: Computational tools for comparative genomics. Nucleic Acids Res..

[54] Katoh K., Standley D.M. (2013). MAFFT Multiple Sequence Alignment Software Version 7: Improvements in performance and usability. Mol. Biol. Evol..

[55] Rozas J., Ferrer-Mata A., Sanchez-DelBarrio J.C., Guirao-Rico S., Librado P., Ramos-Onsins S.E., Sánchez-Gracia A. (2017). DNASP 6: DNA sequence polymorphism analysis of large data sets. Mol. Biol. Evol..

[56] Lanfear R., Frandsen P.B., Wright A.M., Senfeld T., Calcott B. (2017). PartitionFinder 2: New Methods for Selecting Partitioned Models of Evolution for Molecular and Morphological Phylogenetic Analyses. Mol. Biol. Evol..

[57] Ma P., Zhang Y., Zeng C., Guo Z., Li D. (2014). Chloroplast phylogenomic analyses resolve deep-level relationships of an intractable bamboo tribe Arundinarieae (Poaceae). Syst. Biol..

[58] Stamatakis A. (2014). RAxML version 8: A tool for phylogenetic analysis and post-analysis of large phylogenies. Bioinformatics.

[59] Ronquist F., Teslenko M., Mark P.V.D., Ayres D.L., Darling A., Höhna S., Larget B., Liu L., Suchard M.A., Huelsenbeck J.P. (2012). MrBayes 3.2: Efficient Bayesian phylogenetic inference and model choice across a large model space. Syst. Biol..

[60] Rambaut A., Drummond A.J., Xie D., Baele G., Suchard M.A. (2018). Posterior summarization in Bayesian phylogenetics using Tracer 1.7. Syst. Biol..

[61] Wicke S., Schneeweiss G.M., DePamphilis C.W., Muller K.F., Quandt D. (2011). The evolution of the plastid chromosome in land plants: Gene content, gene order, gene function. Plant Mol. Biol..

[62] Zhang Y.T., Huang J., Song J., Lin L.M., Feng R.X., Xing Z.B. (2018). Structure and variation analysis of chloroplast genomes in Fagaceae. Bull. Bot. Res..

[63] Wu Z.H., Gui S.T., Quan Z.W., Pan L., Wang S.Z., Ke W.D., Liang D.Q., Ding Y. (2014). A precise chloroplast genome of *Nelumbo nucifera* (Nelumbonaceae) evaluated with Sanger, Illumina MiSeq, and PacBio RS II sequencing platforms: Insight into the plastid evolution of basal eudicots. BMC Plant Biol..

[64] Chen Y., Hu N., Wu H. (2019). Analyzing and characterizing the chloroplast genome of *Salix wilsonii*. BioMed Res. Int..

[65] Li Y., Lv G.H., Zhang X.N., He X.M. (2017). Chloroplast genome structure and variation analysis of Brassicaceae species. Acta Bot. Boreali-Occident. Sin..

[66] Drescher A., Ruf S., Calsa T.J., Carrer H., Bock R. (2000). The two largest chloroplast genome-encoded open reading frames of higher plants are essential genes. Plant J..

[67] Wang R., Cheng C., Chang C., Wu C., Su T., Chaw S. (2008). Dynamics and evolution of the inverted repeat-large single copy junctions in the chloroplast genomes of monocots. BMC Evol. Biol..

[68] Mardanov A.V., Ravin N.V., Kuznetsov B.B., Samigullin T.H., Antonov A.S., Kolganova T.V., Skyabin K.G. (2008). Complete sequence of the duckweed (*Lemna minor*) chloroplast genome: Structural organization and phylogenetic relationships to other angiosperms. J. Mol. Evol..

[69] Wang X.R., Tang H.R., Duan J., Li L. (2008). A comparative study on karyotypes of 28 taxa in *Rubus* sect. Idaeobatus and sect. Malachobatus (Rosaceae) from China. J. Syst. Evol..

[70] Sun Y.X., Moore M.J., Zhang S.J., Soltis P.S., Soltis D.E., Zhao T.T., Meng A.P., Li X.D., Li J.Q., Wang H.C. (2016). Phylogenomic and structural analyses of 18 complete plastomes across nearly all families of early-diverging eudicots, including an angiosperm-wide analysis of IR gene content evolution. Mol. Phylogenetics Evol..

[71] Cheon K., Kim K., Yoo K. (2017). The complete chloroplast genome sequences of three *Adenophora* species and comparative analysis with Campanuloid species (Campanulaceae). PLoS ONE.

[72] Xue S., Shi T., Luo W., Ni X., Iqbal S., Ni Z., Huang X., Yao D., Shen Z., Gao Z. (2019). Comparative analysis of the complete chloroplast genome among *Prunus mume*, *P. armeniaca*, and *P. salicina*. Hortic. Res..

[73] Downie S.R., Jansen R.K. (2015). A comparative analysis of whole plastid genomes from the Apiales: Expansion and contraction of the inverted repeat, mitochondrial to plastid transfer of DNA, and identification of highly divergent noncoding regions. Syst. Bot..

[74] Bi Y. (2017). Comparative Chloroplast Genomics of the Genus Lilium.

[75] Dong W., Liu J., Yu J., Wang L., Zhou S. (2012). Highly variable chloroplast markers for evaluating plant phylogeny at low taxonomic levels and for DNA barcoding. PLoS ONE.

[76] Wang Y. (2011). Relationships among *Rubus* (Rosaceae) Species Used in Traditional Chinese Medicine. Master’s Thesis.

[77] Yang H.Y. (2016). Phylogenetic Inference in Chinese Rubus L. Based on rpl20–rps12 Sequences.

[78] Pang X., Liu C., Shi L., Liu R., Liang D., Li H., Cherny S.S., Chen S.L. (2012). Utility of the *trnH–psbA* intergenic spacer region and its combinations as plant DNA barcodes: A meta-analysis. PLoS ONE.

[79] Applequist W.L., Wallace R.S. (2002). Deletions in the plastid *trnT–trnL* intergenic spacer define clades within Cactaceae subfamily Cactoideae. Plant Syst. Evol..

[80] Jaramillo M.A., Callejas R., Davidson C., Smith J.F., Stevens A.C. (2008). A phylogeny of the tropical genus *Piper* using its and the chloroplast intron *psbJ–petA*. Syst. Bot..

[81] Zhang W. (2000). Phylogeny of the Grass Family (Poaceae) from *rpl16* Intron Sequence Data. Mol. Phylogenetics Evol..

[82] Dong W., Xu C., Li C., Sun J., Zuo Y., Shi S., Cheng T., Guo J., Zhou S. (2015). *Ycf1*, the most promising plastid DNA barcode of land plants. Sci. Rep..

[83] Okada A., Kikuchi S., Hoshino Y., Kunitake H., Mimura M. (2020). Phylogeny and trait variation of Japanese *Rubus* subgenus *Ideaobatus*. Sci. Hortic..

[84] Yan W., Qing C., Haoru T., Xiaorong W. (2016). Phylogeny of Chinese Rubus (Rosaceae) Based on Nuclear Internal Transcribed Spacer (ITS).

[85] Li W.L., He S.A. (2001). Taxonomic revision on several taxa in the genus *Rubus* (Rosaceae). Bull. Bot. Res..

[86] Wang X., Tang H., Zhang H., Zhong B., Xia W., Liu Y. (2009). Karyotypic, palynological, and RAPD study on 12 taxa from two subsections of section *Idaeobatus* in *Rubus* L. and taxonomic treatment of *R. ellipticus*, *R. pinfaensis*, and *R. ellipticus* var. obcordatus. Plant Syst. Evol..

