# Supplementary figures and images for: Comparative Analysis of Complete Chloroplast Genomes of Rubus in China: Hypervariable Regions and Phylogenetic Relationships
Source: Genes (Basel). 2024 May 31;15(6):716. doi: 10.3390/genes15060716 (PMC11202638; doi:10.3390/genes15060716)

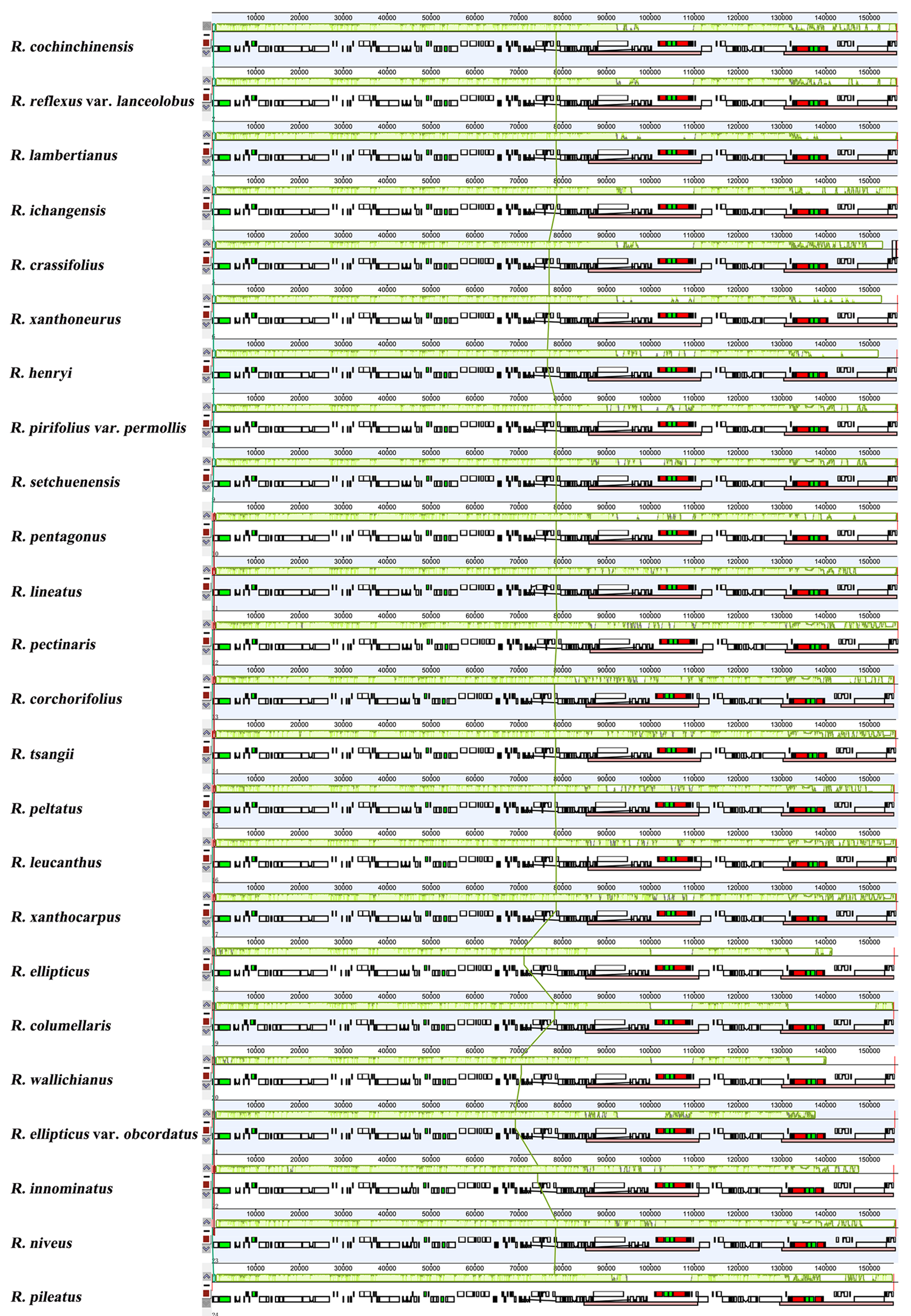

Supplement: Supplementary file 1 [file genes-15-00716-s001.zip › Figure S1.tiff]
